# Improved Strength and Heat Distortion Temperature of Emi-Aromatic Polyamide 10T-co-1012 (PA10T/1012)/GO Composites via In Situ Polymerization

**DOI:** 10.3390/molecules28041960

**Published:** 2023-02-18

**Authors:** Yanchao Dong, Pingli Wang, Zhonglai Ren, Tianyuan Liu, Zhichao Zhen, Bo Lu, Fei Li, Junhui Ji

**Affiliations:** 1 National Engineering Research Center of Engineering Plastics and Ecological Plastics Technical Institute of Physics and Chemistry, Chinese Academy of Sciences, Beijing 100190, China; 2 School of Future Technology, University of Chinese Academy of Sciences, Beijing 100049, China

**Keywords:** in situ polymerization, semi-aromatic polyamide, GO, heat deformation temperature

## Abstract

In this paper, an effective method for preparing poly (p-phenylene terephthalamide) -co- poly (dodecanedioyl) decylamine (PA10T/1012)/graphene oxide (GO) composites by pre-dispersion and one-step in situ polymerization was proposed for the first time. During the process of polycondensation, the condensation between the terminal amino groups of PA10T/1012 chains and the oxygen-containing functional groups of GO allowed nylon to be grafted onto graphene sheets. The effects of polymer grafting on the thermal and mechanical properties of (PA10T/1012)/GO composites were studied in detail. Due to the interaction between PA10T/1012 grafted graphene sheets and its matrix, GO is well dispersed in the PA10T/1012 matrix and physically entangled with it, forming a cross-linked network structure of polymer bridged graphene, thus obtaining enhanced tensile strength, tensile modulus and impact strength. More importantly, benefiting from the cross-linked network structure, the heat distortion temperature (HDT) of the composite is greatly increased from 77.3 °C to 144.2 °C. This in situ polycondensation method opens a new avenue to prepare polycondensate graphene-based composites with high strength and high heat distortion temperatures.

## 1. Introduction

Because of its light weight, good wear resistance, good chemical stability and high impact strength, polymer composites have become a new trend in replacing steel with plastics. Graphene has attracted great attention due to its unique structure and remarkable chemical and physical properties [1,2,3,4,5,6], and is widely used in various fields, such as photoelectric, biomedical, sensors, electronics and supercapacitors. Graphene oxide (GO) is one of the most important derivatives of graphene. Because its surface has a large specific surface area and a large number of hydroxyl, carboxyl and other functional groups [1,5,7,8,9], these characteristics make it an ideal choice for polymer modified fillers to enhance performance or functionalize. So far, a large number of researchers have used PA6, PA12 and other aliphatic nylon to prepare nylon/GO composites through melt blending solvent evaporation or in situ copolymerization [4,10,11,12,13,14,15], which has good mechanical properties, high wear resistance and relatively low cost. Some of them have been industrialized.

PA6 and PA12 are typical aliphatic polyamide materials. As representative varieties of engineering plastics, their melting points generally do not exceed 260 °C, their tensile strength is lower than 60 Mpa, and their heat deformation temperature is lower than 80 °C. However, with the upgrading of traditional industries and the development of emerging technologies, more stringent requirements are placed on the strength and heat resistance of engineering plastics. In this case, since semi-aromatic polyamides combine aliphatic segments with aromatic segments, and has both good processability of aliphatic polyamides and excellent performance of aromatic polyamides [16,17,18], it has become a popular subject in current research. Polyamides contain a large number of aromatic groups, which cause semi-aromatic polyamides to show heat resistance (Tm ≥ 290 °C) which is close to that of fully aromatic polyamide; and high tensile strength (above 80 Mpa). In addition, it also has properties such as high temperature dimensional stability and water absorption reduction. Meanwhile, the existence of a large number of methylene groups in the molecular chain makes the semi-aromatic polyamide exhibit good fluidity, which can be melt-extruded and injection-molded. In the past several decades, semi-aromatic polyamides have been widely used in automobile manufacturing, electronic appliances, and other fields, and have gradually become the most important material in special engineering plastics, such as poly (p-phthaloyl decanediamine) (PA10T) [19,20,21], poly (p-benzoyl dodecyl diamine) (PA12T) [22,23,24], and some copolymers, such as poly (hexamethylene terephthalamide)-copolymer (hexamethylene adipamide) (PA6T/66) and poly (hexamethylene terephthalamide)-copolymer caprolactam (PA6T/6) [25,26]. Therefore, researchers and enterprises have begun to shift their focus from aliphatic nylon composites to semi-aromatic nylon composites. However, the modification of semi-aromatic polyamides has the following two difficulties. First, semi-aromatic polyamides are difficult to dissolve in organic solvents, and it is difficult to carry out solvent blending. Second, limited by the unique polymerization conditions of semi-aromatic polyamides, common synthesis methods of semi-aromatic polyamides, such as melt polycondensation and interfacial polycondensation, make it unsuitable for in situ polymerization. Therefore, the most common modification method for semi-aromatic polyamide modification at present is melt blending. Zhu et al. [27] added 20 wt% of antistatic agent (AA) and 30 wt% of glass fiber (GF) to PA10T matrix by one-step melt blending. They found that the tensile strength of PA10T/AGF composites increased from 66.35 to 88.55 MPa, the electrical resistance decreased from 900 MΩ to 100 MΩ, and the antistatic properties were greatly improved. However, melt blending often has a poor dispersion effect and requires more fillers to achieve the expected result. Therefore, how to effectively disperse fillers is an urgent problem to be solved in the modification of semi-aromatic polyamides.

As we know, the dispersion of nano fillers in a polymer matrix is an important factor affecting the comprehensive properties of composites. The self-aggregation tendency of graphene sheets seriously hinders the efficiency of optimizing graphene oxide reinforced nanocomposites. In order to reduce the self-aggregation tendency of graphene sheets, solvent dispersion, surface modification and in situ polymerization are widely used in the laboratory, and good results have been achieved in earlier reports [28,29]. Among them, the surface polymer modification of graphene oxide has been widely used [13,25], and various surface grafting (grafting or grafting) methods have been further studied because of their favorable dispersion and strong interface with the matrix. As a result, improved performance is achievable using extremely small amount of polymeric graphene oxide due to the large interfacial areas and significant load transfer from polymer to fillers.

In this paper, we developed an effective method for the preparation of PA10T/1012/GO composites based on carboxylated GO and PA10T/1012 in situ polymerization for the first time. PA10T/1012 is a kind of semi-aromatic copolyamide that is produced by introducing flexible dodecanedioic acid segment into the main chain of PA10T. As a result, the processability and toughness of the copolyamide was improved, but the strength and thermal deformation temperature of the material decreased. In this study, GO was creatively introduced into the polymer synthesis process, and PA10T/1012/GO composites were prepared in a reaction kettle by pre-dispersion and in situ polymerization and one-pot reaction. It is hoped that the dispersion of GO in a polymer matrix can be improved by the in situ chemical reaction between the GO and polymer, highlighting the role of GO in improving the performance of composite materials. Firstly, GO was premixed with deionized water by ultrasonic dispersion. A pre-mixed aqueous suspension can disperse GO uniformly, and can be used as the reaction medium for the preparation of PA10T/1012 without using organic solvents, which makes the polymerization process green and pollution-free. Secondly, the aqueous suspension of dodecane, dodecanedioic acid, terephthalic acid, GO and additives were added into a high-temperature autoclave, GO was uniformly dispersed in PA salt by mechanical stirring, and the PA10T/1012/GO composite was prepared by in situ polymerization. The mechanical properties, crystallization behavior, thermo-mechanical properties and thermal properties of the prepared PA10T/1012/GO nanocomposites were systematically studied. The results showed that the mechanical and thermal properties of the prepared composites were significantly improved, and the thermal deformation temperature of the material was greatly increased, which is one of the key performance indicators that determines the service temperature of the material. This method can provide a reference for the design and preparation of high-performance semi-aromatic polyamides.

## 2. Results and Discussion

### 2.1. Structure Characterization of PA10T/1012-Grafted Graphene Sheets

**Figure 1:**
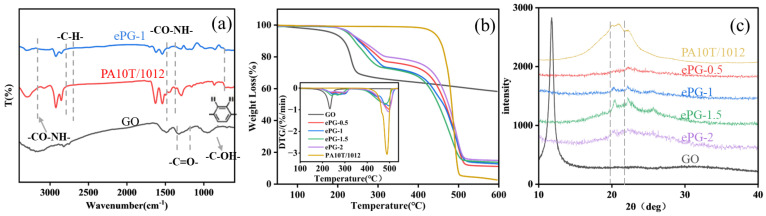
Characterization of GO, PA10T/1012 and ePG (**a**) FTIR spectra; (**b**) TGA and DTG curves; (**c**) XRD patterns.

The presence of PA10T/1012 molecular chains on graphene sheets was detected by FTIR. Figure 1a shows the structural features of GO, PA10T/1012 and ePG-1. We can easily find that GO has typical absorption peaks around 1056 cm^−1^, 1428 cm^−1^ and 1585 cm^−1^, corresponding to the stretching vibration of C-OH, and C=O vibration on carboxyl and carbonyl, respectively [30], which indicated the presence of -OH and -COO- groups on the surface of graphene sheets. For the PA10T/1012 sample, the absorption peaks appeared at 858 cm^−1^ (out-of-plane bending vibration to the C-H on the substituted benzene ring), 1542 cm^−1^, 1624 cm^−1^ (amide-II, amide-I absorption bands) and 3305 cm^−1^ (typical N-H tensile vibration), which indicated that terephthalic acid completely reacted with the amine groups and formed amide groups as expected. Specifically, the C-H tensile vibration and -CO-NH- vibration appeared at 3305 cm^−1^, and the -COO- group was replaced by the absorption bands of Amide-II and Amide-I, which indicated that the PA10T/1012 molecular chain effectively spread into the graphene sheets.

**Figure 2:**
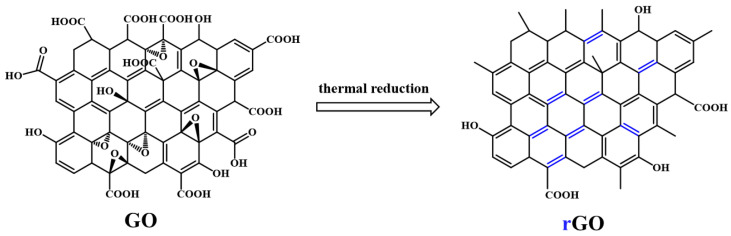
GO is thermally reduced to rGO at high temperature (The blue bonds represent restored conjugated bonds through high-temperature reduction).

We determined the heat decomposition temperatures of the of the obtained ePG samples and GO by Thermogravimetric Analysis (TGA). As shown in Figure 1b, the mass loss (about 34%) of GO in the range of 120–320 °C is mainly due to the removal of oxygen-containing functional groups of epoxy and hydroxyl groups and the decarboxylation of carboxyl groups. The weight loss below 120 °C is the result of water evaporation in the GO sheets. For ePG samples, the figure shows a typical two-stage thermal degradation. The first stage is at 200–320°C, corresponding to the thermal reduction of GO oxygen-containing functional groups, the specific reaction process is shown in Figure 2, and the second stage is at 320–510 °C [31], corresponding to the PA10T/1012 chain segment grafted on GO. It can be seen from the DTG curve in Figure 1b that the temperature of the second stage of the ePG sample to reach the maximum degradation rate is around 485°C, which is almost the same as the temperature of PA10T/1012 to reach the maximum degradation rate. According to the DTG curve, it can be found that the decomposition rate of ePG samples is slower than that of GO, which means that the thermal stability of ePG in this temperature range is better than that of GO. Moreover, the weight loss curve of ePG samples remained basically unchanged below 200°C, which indicated that some unstable functional groups of GO had been transformed into stable bonds or were decomposed during the high-temperature polycondensation process. In this in situ polymerization, GO materials were partially thermally reduced to graphene while grafting PA10T/1012 chains. However, below 600 °C, the carbon residue value of ePG-0.5, ePG-1, ePG-1.5 and ePG-2 gradually increased, indicating that the contents of grafted polymers decreased with the increase of GO addition.

Figure 1c shows the XRD patterns of ePG. A characteristic diffraction peak was detected at 11.2° of GO due to the oxygen-containing groups on the graphite sheets increasing the interlayer distance of the graphene oxide sheets. Compared with GO, the diffraction peak of ePG at 11.2 disappeared, indicating that the thermal reduction of GO occurred. A new peak at 20.1° and 22.3° indicated that PA10T/1012 chains were successfully grafted onto the surface of ePG [10].

**Figure 3:**
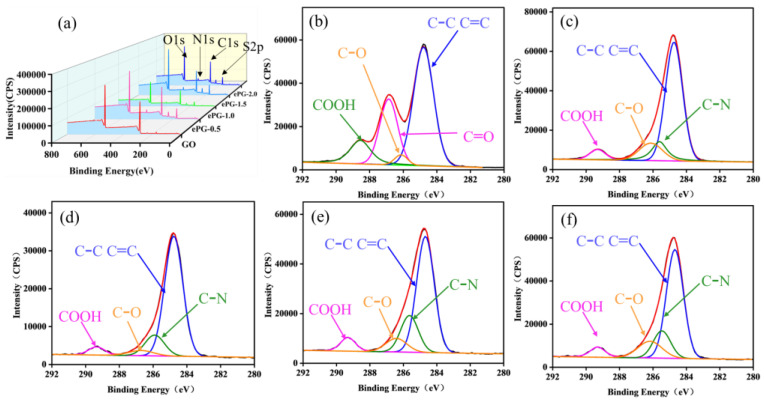
XPS spectra of (**a**) GO, ePG 0.5-2; and C1s peak-fitting curves of (**b**) GO, (**c**) ePG-0.5, (**d**) ePG-1, (**e**) ePG-1.5 and (**f**) ePG-2.

In addition, the XPS results (Figure 3a) show that there is a C1s peak at 284.5 eV, an O1s peak at 533.4 eV, no nitrogen peak, and an N1s peak at 401 eV for the ePG sample. The peak at 171.2 eV corresponds to S2p, which is due to the residual sulfuric acid in the ePG composite. As shown in Figure 3b, the graphene oxide samples have C-C, C=C (sp^2^C) peaks at 284.5 eV, C-O peaks at 286.2 eV, C=O peaks at 287.4 eV, and C=O peaks at 289.0 eV [32,33]. These groups endow graphene oxide sheets with a polarized surface and ensure their good dispersion in aqueous solution and good connection with polymers. Comparing GO and ePG (Figure 3c–f), the C-O was replaced by the C-N peak at 285.7 eV, indicating the formation of -CO-NH- and the thermal reduction of GO (C-OH bond breaking) at this temperature [34]. In addition, as shown in Table 1, compared with GO samples, the ratio of nitrogen and oxygen in ePG increases, and the relative atomic concentration of nitrogen and carbon (N/C) and that of oxygen and carbon (O/C) is higher, fully verifying that the PA10T/1012 chain is efficiently grafted onto graphene nanosheets.

### 2.2. Structure and Intrinsic Viscosity of SPA and MPA Composites

#### 2.2.1. Structural Characterization of SPA and MPA Composites

**Figure 4:**
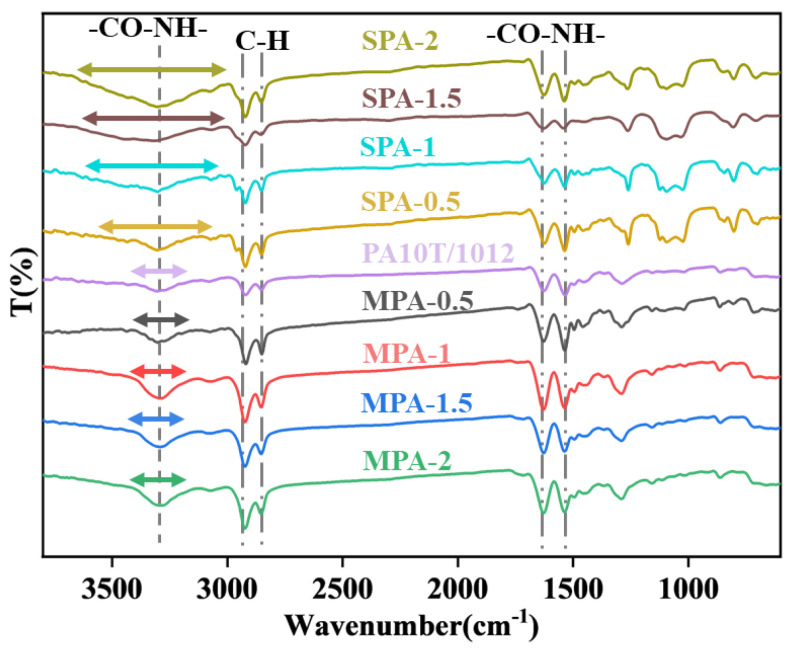
FTIR spectra of MPA composites and SPA composites.

Figure 4 shows the FTIR spectra of SPA and MPA composites. The SPA/MPA composites show almost the same infrared characteristic absorption peaks as PA10T/1012, with absorption peaks at 3305, 2924, 2853, 1627, 1536 and 1288. Among them, the absorption peaks of N-H stretching vibration, C-H stretching vibration and C-H stretching vibration are located at 3305, 2924 and 2853 cm^−1^ [35], respectively. The absorption peaks at 1627, 1536 and 1288 cm^−1^ belong to the amide I band (C=O stretching vibration absorption peak), amide II band (C-N stretching vibration and N-H bending vibration absorption peak), amide III band (C-N stretching vibration and C-H bending vibration absorption peak), and amide V band (N-H in-plane bending vibration absorption peak), respectively. Compared with the MPA composites, the N-H stretching vibration absorption peak of the SPA composites at 3305 cm^−1^ becomes stronger and the band is significantly broadened. This is because of the enhanced interfacial bonding between GO and PA10T/1012 matrix due to grafting, and the oxygen-containing groups on the surface of GO easily form hydrogen bonds with PA10T/1012 molecular chains. Hydrogen bonds weaken the electron clouds and chemical bonds, elongating the normal N-H covalent bond length, diversifying the characteristic frequency, and broadening the absorption peak. On the other hand, with the increase of GO contents, the number of intermolecular hydrogen bonds increases, and the characteristic peak intensity also increases.

#### 2.2.2. Characterization of Intrinsic Viscosity and Crosslinking Degree

**Figure 5:**
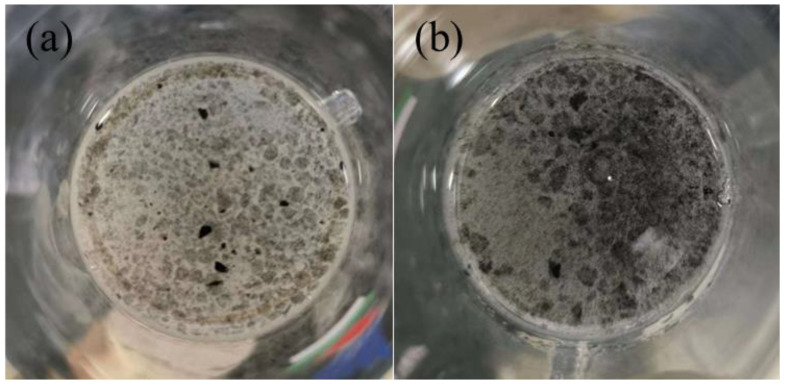
The swelling phenomenon of SPA composites in concentrated sulfuric acid: (**a**) SPA-1.0 wt% GO, (**b**) SPA-2.0 wt% GO.

**Figure 6:**
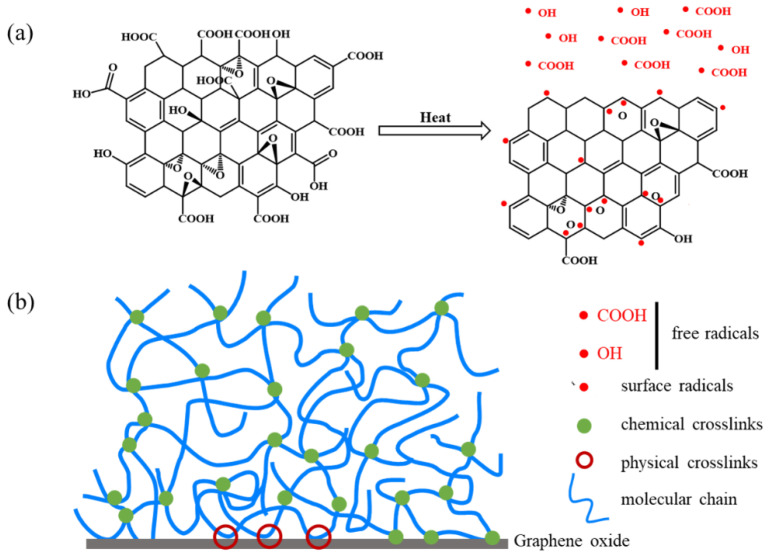
A schematic description of the crosslinking mechanism of GO. (**a**) GO contains oxygenic groups such as hydroxyls that may undergo homolytic cleavage at high temperature; (**b**) Schematic diagram of crosslinking of graphene oxide surface.

In this paper, GO and PA10T/1012 are introduced for copolymerization. The introduction of GO makes the polymer crosslinked to varying degrees, and the degree of crosslinking can be characterized by the test of gel content. Concentrated sulfuric acid is a good solvent for PA10T/1012 and can dissolve PA10T/1012. However, the cross-linked part in the sample cannot be dissolved by concentrated sulfuric acid to cause swelling, as shown in Figure 5. Table 2 shows the intrinsic viscosity of the sample ([ƞ]) and gel content (G). With the increase of GO content, the gel content of the sample gradually increases, indicating that the degree of crosslinking of the sample increases with the increase of GO content. The decomposition of oxygen-containing groups on graphene oxide produces two kinds of free radicals, one is the free radical on the cleavage group, and the other is limited to the GO surface, as shown in Figure 6a. These two free radicals can induce the crosslinking of the polyamide, which is similar to the crosslinking mechanism of peroxide. As shown in Figure 6b, free radicals on the cleavage group can diffuse out of graphene oxide, leading to the cross-linking of polyamide molecular chains; however, the free radicals on graphene oxide are localized, leading to the interface crosslinking between the graphene oxide and the polyamide molecular chain. In addition to the chemical crosslinking induced by free radicals, there is also physical crosslinking (empty ring) due to polymer adsorption on graphene oxide [36]. Intrinsic viscosity is normally usually used to estimate the molecular weight of polyamide. As can be seen from Table 2, the molecular weight of the SPA sample decreases with the increase of GO mass fraction, from 11, 321 g/mol of pure PA10T/1012 to 7554 g/mol of SPA-2.0 wt% composites. This is because in the polycondensation process, the excessive carboxyl groups on graphene oxide sheets inevitably destroy the stoichiometric balance between carboxylic acids and amino acids in the reaction system, and the polymer chains graft onto graphene sheets, while at the same time, the extension of the active chain ends is terminated. Therefore, the higher feed ratio of GO to monomer, the more carboxylic acid contents of GO, the smaller the relative molecular weight of grafted polymer chains, and the smaller the mass fraction of grafted polymers.

### 2.3. X-ray Diffraction Analysis

**Figure 7:**
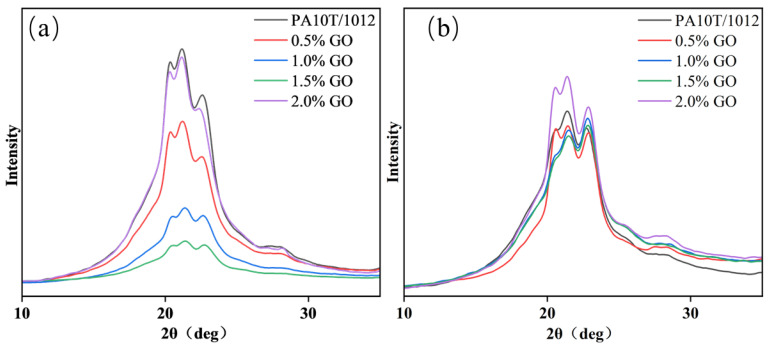
XRD spectra: (**a**) SPA composites; (**b**) MPA composites.

In this work, the crystal structure and crystallization behavior of SPA and MPA composites were studied using X-ray diffraction, as shown in Figure 7. Firstly, the introduction of GO did not change the crystal form or crystal structure of PA10T for SPA or MPA. This mainly shows in the same crystal structure as PA10T/1012, the same peak position, and the diffraction peaks are located at 2θ = 20.1°, 21.0° and 22.3° [37], respectively. Secondly, with GO as a rigid filler, its introduction will occupy the free volume of the molecular chain and will restrict the movement of the polymer chain, so it will have an impact on crystallinity. This situation is manifested in the XRD results as a weakening of the diffraction peak intensity. Compared to the SPA and MPA composites, when the GO content is the same, the SPA has a greater effect on the crystallinity and the decline trend is more obvious. This is because GO in SPA samples is connected with PA10T/1012 through chemical bonds, while MPA only restricts the movement of PA10T through physical actions. Obviously, SPA has a stronger hindering effect on the movement of the PA10T/1012 molecular chain. However, when the content of GO increases to a certain extent, the effect of hindering movement is weakened perhaps due to the partial aggregation of GO, so the diffraction peaks become stronger again. This will be further verified in the subsequent DSC analysis of the crystallization process of the materials.

### 2.4. Thermal Analysis

#### 2.4.1. Differential Scanning Calorimeter Analysis

**Figure 8:**
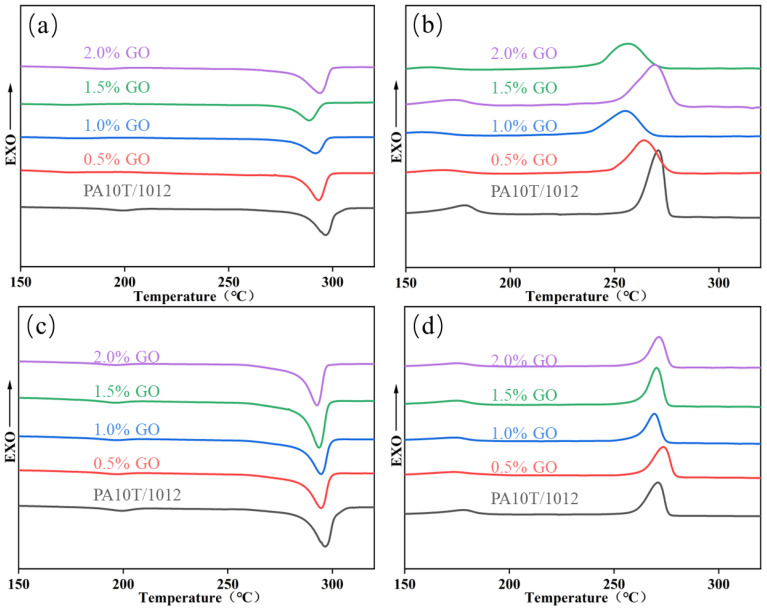
Differential scanning calorimetry (DSC) thermograms: SPA composites (**a**) Secondary heating curve; (**b**) Cooling curve; MPA composites (**c**) Secondary heating curve; (**d**) Cooling curve.

The authors analyzed the effect of GO on the melt crystallization behavior of PA10T/1012/GO composites through DSC experiments. Figure 8a–d show the DSC curves of the SPA and MPA composites, from which the melting temperature (Tm) of the samples can be derived. The data of thermal properties are collected in Table 3.It can be seen from Figure 8a,b that the overall changes in T_m_ and T_c_ of the two composites are not significant. In the previous discussion about XRD, it is known that the crystal structure of the composites does not change, and the change of melting point mainly comes from the change in the crystallinity of PA10T/1012. This is mainly because the addition of GO, a rigid filler, destroys the symmetry and regularity of the PA10T/1012 molecular chain. The more GO is added, the more the regularity of PA10T/1012 is destroyed, and the more the melting temperature drops. In addition, during the in situ polymerization of the composites, the carboxylic acid on the GO sheet terminated the active chain, and the relative molecular mass of the polymer decreased with the increase of GO content, which led to a decrease of melting temperature. Due to the grafting and crosslinking of GO and polyamide copolymerization, the binding effect of GO on molecular chains in SPA composites is stronger than that of pure blending, and the melting point of SPA is changing more significantly than that of MPA as a whole.

The crystallinity can be determined by the ratio of melting enthalpy to that of fully crystallized samples. Here, assuming that the melting enthalpy of all samples corresponding to samples with fully crystallized crystal regions are same, the crystallinity can be approximately compared by comparing _Δ_H_m_. It can be seen from Table 3 that with the increase of GO addition, for SPA composites, the melting enthalpy (_Δ_H_m_) of the sample gradually decreases for the SPA composite. On the one hand, the decrease in crystallinity is attributed to the fact that with the increase of GO content, Nylon molecular segments are blocked in the process of movement, so it is difficult to grow in a radial symmetrical and regular arrangement, which hinders the crystallization process. On the other hand, the polyamide molecular chain of the SPA composite is grafted with GO sheets, which can more effectively hinder crystallization than the simple physical action of MPA composites. In addition, the crosslinking induced by GO in the polymerization process also limits the movement of the polyamide molecular chain to some extent.

When the GO content is 2.0 wt%, the _Δ_H_m_ of the SPA composite increases to 40.85 J/g, which is close to that of pure PA10T/1012. All of these are caused by agglomeration. Compared with SPA composites, the Tm of MPA composites is relatively higher because of its higher polymer molecular weight and crystallinity compared to SPA composites. This is consistent with the change of crystallinity observed by XRD.

#### 2.4.2. Thermal Mechanical Properties

**Figure 9:**
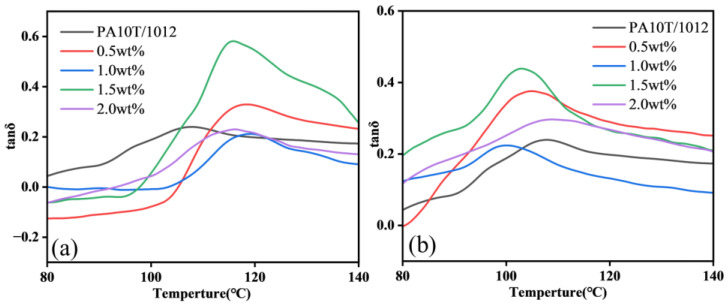
Dynamic damping factor (tanδ) curves (**a**) SPA composites, (**b**) MPA composites.

Figure 9 shows the tanδ curve of the PA10T/1012 and SPA composites. The peak at the low temperature zone is caused by α relaxation, and the peak temperature at this point is generally considered as the glass transition temperature T_g_, which is an important parameter to measure the heat resistance. As shown in Figure 9a, the T_g_ of SPA composites is obviously increased. Although the crystallinity of SPA composites is decreased caused by the introduction of GO, the PA chain is crosslinked. The effect of interfacial interaction between the polymer and graphene oxide surface on the dynamic thermo-mechanical properties of SPA composites can be qualitatively understood based on the multi-scale relaxation properties of the polymer. As mentioned earlier, the crosslinking of the PA molecular chain not only occurs in the polymer matrix because of the diffusion of free radicals, but also occurs on the surface of graphene oxide because of the localization of free radicals. With the chemical cross-linking initiated by free radicals on interface, the physical interaction of CH-p leads to the polymer being adsorbed on the surface of the graphene oxide, as illustrated in Figure 6. Furthermore, both chemical and physical interactions may render a surface coated with polymers forming a de Gennes’ carpet-like structure, in which the effective crosslinking density increases to the substrate surface, as illustrated in Figure 6b. The polymer network with higher cross-linking density reduces the free volume of molecular chains and the chain mobility, which leads to a higher glass transition temperature (T_g_). Therefore, SPA composites have higher T_g_, which also indicates that the introduction of GO causes the heat resistance of the materials to increase.

As shown in Figure 9b, the T_g_ of MPA composites shows a downward trend with the increase of GO contents. Compared with the well-dispersed SPA composites, GO agglomerations increase the free volume among polymer chains, and reduce the effective interface and crystallinity, which in turn leads to the decrease of T_g_. As shown in Table 4, the tanδ of the composite materials is significantly higher than that of PA10T/1012, indicating that the energy dissipation capacity of the materials is increased, which is consistent with the observed increase in the impact strength of samples. This indicates that the toughness of the sample is improved. The tanδ of SPA composites is larger than that of MPA composites, which is because the grafting is beneficial to the uniform dispersion and strong binding with the matrix interface.

#### 2.4.3. Thermogravimetric Analysis

**Figure 10:**
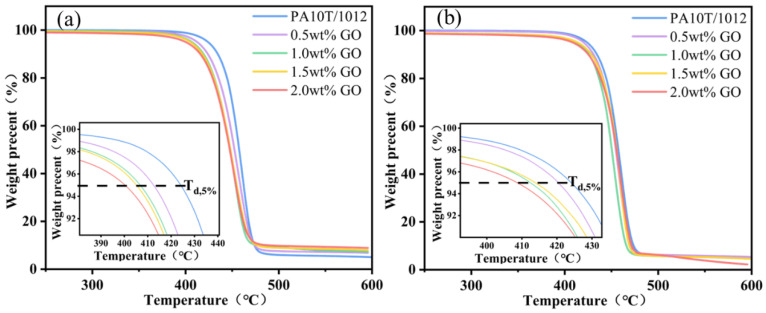
Thermogravimetric (TG) curves: (**a**) SPA composites; (**b**) MPA composites.

The authors analyzed the effect of GO on the thermal stability of the PA10T/1012/GO composite by TGA experiment. Figure 10 shows the TGA curves of SPA and MPA composites. It can be seen from Figure 10 that the initial decomposition temperature of SPA and MPA composites gradually decreases with the increase of GO content, indicating that the thermal stability of PA10T/1012 is reduced by doping GO into the PA matrix. This may be due to the easy decomposition of carboxyl or hydroxyl groups on the GO nanosheets, which would catalyze the degradation of PA10T/1012 at high temperature, thereby reducing the thermal stability of PA10T/1012. In a TGA test, it was obviously detected that the SPA composites exhibited a lower decomposition threshold temperature compared with the MPA composites, indicating that the molecular weight of PA10T/1012 in the SPA composites was relatively low. This was because the excessive carboxylic acids of GO intensively destroyed the stoichiometric balance [30].

#### 2.4.4. Heat Deflection Temperature

HDT is one of the most important parameters used to judge the heat resistance of plastic products, having a very important practical application value. The factors affecting the heat distortion temperature mainly include the following points. First, for amorphous polymers, the HDT is mainly related to the mobility of molecular chains, and the heat distortion temperature is near the glass transition temperature. Second, for crystallization polymer materials, the amorphous area is small, and the HDT is mainly affected by the crystallization area. When the crystallinity is within a certain range, the HDT increases when the crystallinity increases. Third, adding inorganic fillers or other rigid fillers will hinder the movement of molecular chains, and adding cross-linking modifiers will reduce the mobility of the chain segments. At the same time, when the filler exceeds a certain amount, it can form a rigid network structure which can also improve the heat resistance to a certain extent [38,39].

**Figure 11:**
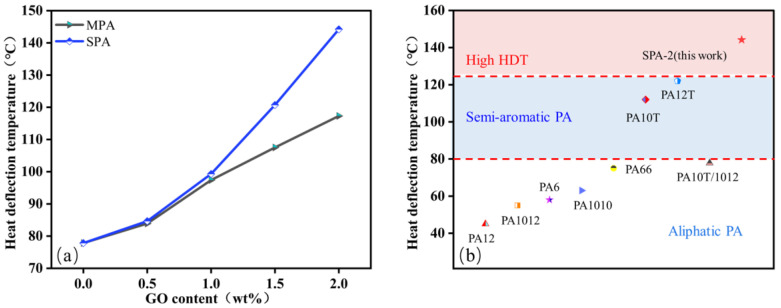
(**a**) Thermal deformation temperature curve of SPA composites and MPA composites; (**b**) Thermal deformation temperatures of SPA composites compared with other polyamides.

Figure 11a shows the HDT curves of SPA and MPA composites. Figure 11b shows the comparison between the heat distortion temperature of SPA-2.0 wt% and other polyamides. The heat distortion temperature of most polyamides is below 80 °C, while the introduction of GO in this work has greatly increased the heat distortion temperature. The heat distortion temperature of SPA-2.0 wt% composites increased to 144.2 °C, which is 85.28% higher than that of PA10T/1012. This is a crucial step-forward for the practical application of this composite. It can be seen from Table 4 that the heat distortion temperature of SPA composites is higher than that of MPA composites, for which there are two reasons.

**Figure 12:**
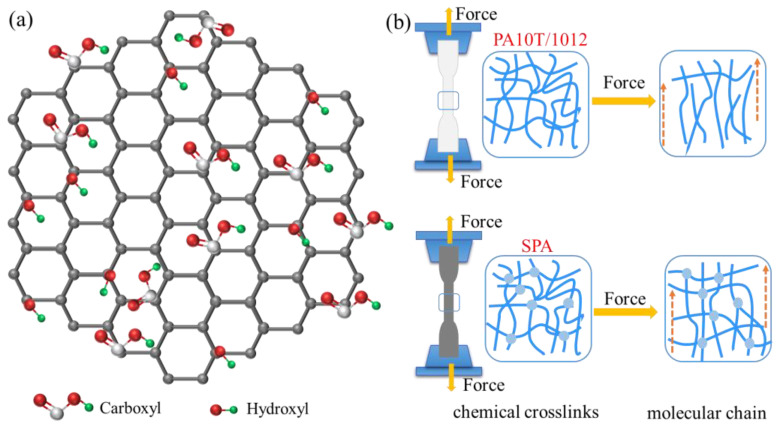
(**a**) Structure model of GO; (**b**) Tensile deformation mechanism.

The first reason is the reinforcement of the rigid filler GO. As shown in Figure 12a, the carbon atoms of GO are connected by s-bonds in the form of sp^2^ hybridization. These s-bonds endow graphene with excellent structural rigidity. Three electrons in the outer layer of the C atom of graphene oxide form a strong s-bond through sp^2^ hybridization, and the angle between two adjacent bonds is 120°. The C-C bond length of graphene is about 0.142 nm; each crystal lattice has three s-bonds; p orbitals of all carbon atoms are perpendicular to the sp^2^ hybridization plane, and they form a delocalized π bond in a side-by-side manner, which runs through the entire graphene [40]. Therefore, graphene can be regarded as an infinite aromatic molecule. Each carbon atom hybridizes with the surrounding carbon atoms to form a regular hexagon, and each hexagonal unit is actually similar to a benzene ring, which makes graphene have excellent structural rigidity.

The second is the crosslinking mechanism of graphene oxide, which is similar to peroxide, that makes the polyamide molecular chain form a cross-linking network centered on graphene. Consequently, the interfacial adhesion between GO and PA was enhanced, hindering the movement of PA chains, which caused the slow relaxation of the PA chains and significantly increased the heat distortion temperature [41,42,43].

### 2.5. Mechanical Properties of PA10T/1012/GO

**Figure 13:**
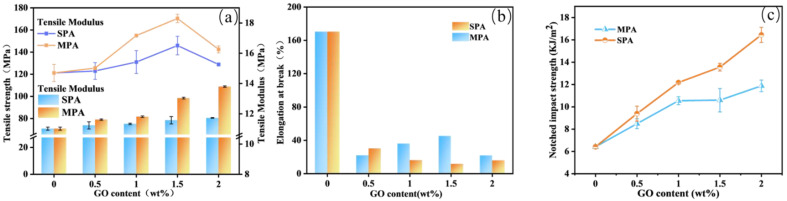
Mechanical parameters of SPA composites and MPA composites: (**a**) Tensile strength and Tensile modulus; (**b**) Elongation at break; (**c**) Notched impact strength.

The mechanical properties of SPA and MPA composites are shown in Figure 13. When the GO concentration increased from 0.0 wt% to 2.0 wt%, the tensile strength, tensile modulus, and impact strength gradually increased. Compared with PA10T/1012, as show in Table 5, when the GO content was 2.0 wt%, the tensile strength, tensile modulus and impact strength of the SPA composites significantly improved by 54%, 23% and 156%, respectively. Based on the comparison of the mechanical properties of SPA and MPA composites, it was found that GO has a more significant reinforcing effect on the composites through in situ polymerization. This enhancement effect is, firstly, due to the uniform dispersion and strong interfacial bonding of GO in the matrix, and the PA molecular chains are successfully grafted onto graphene. The modified surface features make the functionalized graphene oxide sheets uniformly dispersed in SPA composites.

**Figure 14:**
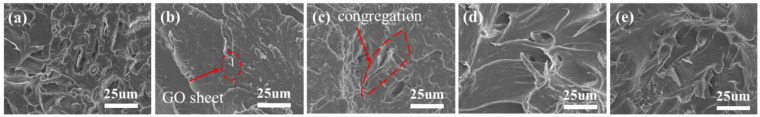
Scanning electron microscopy (SEM) images of tensile section: (**a**) PA10T/1012: (**b**) MPA-1.0 wt% GO: (**c**) MPA-2.0 wt% GO: (**d**) SPA-1.0 wt% GO: (**e**) SPA-2.0 wt% GO.

As can be seen from the cross-sectional image of SPA in Figure 14, it can be determined that there is a smooth fracture surface and no aggregation of GO sheets. The entanglement of grafted long-chain PA10T/1012 with free segments leads to strong interfacial bonding, which is conducive to the transfer of stress from polyamide to graphene oxide sheets and effectively avoids the self-aggregation of GO. However, the fracture surface of MPA composites is rough and has obvious GO agglomeration. In addition, the tensile sections of the SPA composites exhibited a fibrous morphology, indicating that extensive deformation occurred before the tensile fracture. In sharp contrast, the surface morphology of fractured MPA composites shows limited plastic deformation, implying that the fracture behavior is brittle, which is due to the lack of restrictions and implications of chemical crosslinks. However, if the adhesion between the fillers and the matrix is insufficient, extensive delamination usually occurs between the polymer matrix and the fillers [44]. The fracture surface of MPA samples shows obvious delamination, while SPA samples show little plastic deformation, indicating that the interfacial adhesion between the GO and polyamide matrix in SPA composites is enhanced.

Secondly, this occurs because of the crosslinking of PA, as shown in Figure 6b. The crosslinking density decreases as the distance from the GO surface increases, and eventually becomes a constant, which forms a crosslinking network centered on the graphene oxide sheets. With the increase of GO contents, the density of cross-linking points increases, resulting in strong interfacial interactions. And thirdly, it is the result of the formation of hydrogen bonds and amide bonds between the surface oxygen-containing groups of GO and the PA10T/1012 matrix, so that a strong interaction force can be formed between GO and PA10T/1012. As a result, the tensile strength and impact strength of nanocomposites are greatly improved.

In addition, when the GO contents increased from 0.0 wt% to 2.0 wt%, the elongation at the break of nanocomposites decreased from 170.47% to 14.23%. We can see that, with the increase of GO contents, the tensile toughness of copolymer molecular chains decreased. The tensile deformation mechanism is shown in Figure 12b. The formation of this diplomatic linkage structure limits the load transfer between the two phases, so that the materials are not easy to deform and the elongation at the break reduces. On the other hand, the mechanical interlocking characteristic of GO nanosheets reduces the matrix mobility within nanocomposites, thus reducing its ductility when compared to PA10T/1012.

## 3. Experiments

### 3.1. Experimental Reagents

Bio-based 1, 10-diaminodecane (DMD, 98.5%) was purchased from Wuxi Yinda Nylon Co., Ltd. (Wuxi, China). Dodecanoic acid (DDA, 99%) was purchased from Beijing Enokai Co., Ltd. (Beijing, China). Commercially available Terephthalic acid (TPA, 99%) was acquired from XiLong Chemical Co., Ltd. (Guangzhou, China). Graphene oxide (GO, purity 95 wt%, thickness about 3~8 nm, sheet diameter about 10 μm) was purchased from Suzhou Tanfeng Graphene Technology Co., Ltd. (Nanjing, China). Benzoic acid (BA, 99.5%) and sodium hypophosphite (SHP, 99%) were purchased from Xilong Chemical Co., Ltd. (Guangzhou, China). Concentrated sulfuric acid (96%) was purchased from the Beijing Chemical Reagent Company. High-purity nitrogen (N2, 99.999%) was purchased from the Beijing HuanyuJinghui Gas Technology Co., Ltd. (Beijing, China). Deionized water was self-made.

### 3.2. Synthesis of PA10T/1012/GO Composites

#### 3.2.1. PA10T/1012/GO Composites with Different GO Contents Were Prepared by In Situ Polymerization

**Scheme 1:**
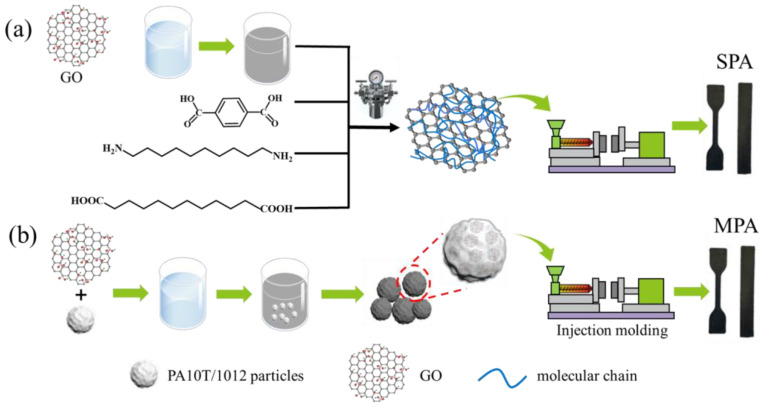
Preparation of PA10T/1012/GO composites by in situ polymerization: (**a**) SPA composites; (**b**) MPA composites.

The route of preparing PA10T/1012/GO composites is shown in Scheme 1a. The specific steps are as follows:

(a) GO was added to deionized water and treated ultrasonically for 1 h to form an aqueous suspension containing less than 2.0% GO.

(b) DMD (1.01 mol, 174.03 g), TPA (0.9 mol, 149.52 g), DDA (0.1 mol, 23.03 g), GO aqueous suspension (500 mL, respectively containing 1.74 g, 3.48 g, 5.22 g, 6.96 g GO), BA (0.01 mol, 1.22 g), and SHP (0.1 wt%, 0.34 g) were added into a stirred autoclave. Then, set the stirring device to 50 rpm, and purge the autoclave five times with N2 for air replacement. Heat the autoclave to 80 °C and keep it for about 1 h to obtain nylon salt. Then, heat the autoclave to 235 °C and keep it for about 1 h. The pressure of the autoclave is kept within 2.5 MPa by discharging steam, and nylon salt is converted into a prepolymer under high temperature and pressure. Then, with the discharge of water vapor, the molecular weight of the prepolymer gradually increased, and the pressure was uniformly reduced to atmospheric pressure over 1 h, and then cooled to room temperature to obtain the PA10T/1012/GO prepolymer.

(c) After pulverization, the prepolymers were put into a vacuum oven. The vacuum oven was evacuated within 20 Pa, heated to 250 °C and held for 4 h. Finally, the vacuum oven was naturally cooled down to room temperature after the polymerization was completed. For a clearer description, the PA10T/1012/GO composite obtained by in situ polymerization will be expressed as SPA in the following discussion. At the same time, when the feeding ratio of GO is 0.5 wt%, 1.0 wt%, 1.5 wt% and 2.0 wt%, respectively, the samples obtained are represented by SPA-0.5 wt% GO, SPA-1.0 wt% GO, SPA-1.5 wt% GO and SPA-2.0 wt% GO composites.

#### 3.2.2. PA10T/1012/GO Composites with Different GO Content Were Prepared by Physical Blending

Similar to SPA above, we abbreviated PA10T/1012/GO composites obtained by physical blending as MPA, and its preparation method is shown in Scheme 1. The specific steps are as follows:

(a) GO was added to anhydrous ethanol and 30 min later was treated ultrasonically to form an ethanol suspension; (b) The PA10T/1012 particles (D90 < 100 *μ*m) prepared in 2.2.1 were added to the ethanol suspension, stirred by magnetic force at room temperature for 1 h, and then heated to 85 °C to remove ethanol; (c) The resulting mixture was transferred to an oven and dried overnight at 80 °C. The content of GO in the mixture is 0.5, 1.0, 1.5 and 2.0 wt%, respectively; (d) the sample was molded by an injection molding machine. When the content of GO is 0.5 wt%, 1.0 wt%, 1.5 wt% and 2.0 wt%, respectively, the samples obtained are represented by MPA-0.5 wt% GO, MPA-1.0 wt% GO, MPA-1.5 wt% GO and MPA-2.0 wt% GO composites.

### 3.3. The Collection of ePG from SPA Composites

In order to verify the chemical reaction between GO and PA10T/1012 in the process of in situ polymerization, the PA10T/1012-grafted graphene sheet (Later expressed as ePG) in SPA was separated by referring the difference of solubility between GO and polymer in concentrated sulfuric acid.

While the GO or GO grafted polymer chain were insoluble in concentrated sulfuric acid, the purified PA10T/1012 was removed by dissolving SPA composites and centrifuging, and with the repeated washing with concentrated sulfuric acid and ethanol, and the final product was a PA10T/1012-grafted graphene sheet. It is worth noting that the aforementioned method cannot completely remove pure GO that does not react with PA10T/1012 in theory, but the characterization of ePG here is more important to confirm the grafting reaction between GO and PA10T/1012, as well as the change of grafting amount. The products separated from SPA-0.5 wt%, SPA-1.0 wt%, SPA-1.5 wt% and SPA-2.0 wt% were identified as ePG-0.5, ePG-1.0, ePG-1.5 and ePG-2, respectively.

### 3.4. Characterization

Fourier transform infrared (FTIR) spectra were recorded on an FTIR spectrophotometer (JASCO FT/IR-6800) by the accumulation of 32 scans with a resolution of 2 cm^−1^.

Characterization of the cross-linking degree: the degree of cross-linking is expressed by the gel content (G) of the sample after swelling. Weigh a certain mass of sample and record it as m_1_. Dissolve the sample in 50 mL 96% concentrated sulfuric acid for 24 h, and then filter it through a G2 core funnel. The swollen part of the sample cannot pass through the core funnel. Slowly pour the filtrate into 1000 mL absolute ethanol, and continuously stir to separate the dissolved part of the sample. After separation, washing, and drying, weigh its mass. It is recorded as m_2_, and the gel content can be calculated by the following formula:

G = [(m_1_ − m_2_)/m_1_] × 100%

Viscosity test: dissolve the dried sample in 96% concentrated sulfuric acid, prepare a polymer solution with a concentration of 0.005 g/mL, and then pass the polymer solution through the sand core funnel (10–15 μm). Filter it into the Ubbelohde viscometer, and place the viscometer in a constant temperature water bath for testing. The temperature of the water bath is ·25 ± 0.05 °C. Each group undergoes three parallel tests. The test error does not exceed ± 0.2 s. The average value of the test results is taken. The efflux time of concentrated sulfuric acid and the polymer solution are recorded as t_0_ and t_1_, respectively. The intrinsic viscosity, recorded as [*η*], was calculated by the Solomon and Ciuta relationship:

$$\left[\eta \right]{=[2\;(\eta }_{\mathrm{sp}}{-\ln\eta }_{r}{)]}^{1/2}/c$$

where *c* (concentration of the polymer solution) is 0.005 g/mL, *η_sp_* (specific viscosity) was calculated by (t_1_/t_0_-1), and *η_r_* (relative viscosity) was obtained by t_1_/t_0_. The Mark-Houwink equation [45] was used to estimate the M_w_ of PA10T/1012 and 10T/1012/GO composites:

$$\left[\eta \right]=3{.824\;\times \;10}^{-4}{\;M}_{w}^{\;0.8159}$$

A scanning electron microscopy (SEM) analysis was adopted to observe the impact of the fractured surface using a JSM-7610FPlus field emission scanning electron microscope.

The X-ray photoelectron spectroscopy (XPS) analyses were conducted with a ESCALAB 250Xi photoelectron spectrometer.

The X-ray diffraction (XRD) patterns of the samples were recorded on a Bruker D8 focus instrument using CuK α radiation in the 2θ range from 5° to 60° at a scanning step of 0.2°/s.

Differential scanning calorimetry (DSC) measurements were performed on a Mettler DSC1 instrument under nitrogen atmosphere. Samples were heated to 340 °C to remove the thermal history and kept at 340 °C for 10 min. The samples were then cooled to 20 °C and were subsequently heated to 340 °C again at a rate of 10 °C/min.

A thermal gravimetric analysis (TGA) was performed on a TA Q50 instrument at a heating rate of 10 °C/min from 25 °C to 600 °C under N2.

A dynamic mechanical analysis (DMA) was performed on a Mettler DMA/SDTA861e instrument operating in shear mode. The samples were prepared by injection molding by Thermo Scientific MiNiJet Pro. Data were recorded from 25 °C to 180 °C with a heating rate of 3 °C/min at a frequency of 1 Hz.

Tensile property test: sample size 25 × 4 × 2 mm^3^, the tensile rate is 5 mm/min. The average value of five parallel tests is taken as the test result.

Heat deflection temperature (HDT) test: reference GB/T1634-2004 test, spline flat, applied bending stress of 1.80 Pa, heating rate of 120 °C/h, test results are taken as the average of three groups of parallel tests.

Impact performance test of simply supported beam: sample size 80 × 10 × 4 mm^3^, the notch is a 2 mm deep V-shaped notch, the test temperature is room temperature, and the test results are taken as the average of five groups of parallel tests.

## 4. Conclusions

In conclusion, the authors prepared PA10T/1012/GO composites through the in situ polymerization of PA10T/1012 in the presence of graphene oxide. The macromolecular chains of PA10T/1012 were efficiently grafted onto GO nanosheets through the condensation reaction between the carboxylic acid groups on the GO surface and the amino groups at the end of the PA10T/1012 chain, accompanied by heat reduction from GO to rGO. The grafted GO nanosheets have good compatibility and strong interfacial interaction with the PA10T/1012 matrix, which is the key factor to improve the mechanical and thermal properties of PA10T/1012/GO composites. The Young’s modulus and tensile strength of the SPA composites are increased to 96.63 MPa and 1807 MPa, respectively, the glass transition temperature is increased from 106.6 °C to 118.97 °C, and the heat deformation temperature is increased from 77.83 °C to 144.2 °C. Compared with SPA composites, MPA composites are inferior in heat resistance and mechanical strength due to their own limitations. Its advantages mainly lie in its simplicity, high efficiency and large-scale application. MPA and SPA composites have high HDT temperature and good mechanical properties, so they can be used in SMT connectors, heat-resistant parts under automobile engines, and in other fields. Among them, SPA composites can be used in higher-end products because of their relatively superior properties.

## Figures and Tables

**Table 1:** Contents of different elements of GO and ePG.

Sample	Elemental Composition			Elemental Composition	
	C (%)	N (%)	O (%)	N/C (×10^−2^)	O/C (×10^−2^)
GO	67.24	1.25	28.79	1.86	42.82
ePG-0.5	52.77	3.87	33.16	7.33	62.84
ePG-1.0	51.32	3.11	32.82	6.06	63.95
ePG-1.5	47.17	3.69	34.81	7.82	73.79
ePG-2	49.98	3.52	38.51	7.04	77.05

**Table 2:** Gel content and intrinsic viscosity data of SPA samples.

GO Content (wt%)	0	0.50	1.00	1.50	2.00
[*η*] (dL/g)	0.774	0.593	0.567	0.563	0.558
*M*_w_ (g/mol)	11, 321	8136	7701	7641	7554
G (%)	0	0.24	0.39	0.64	0.85

**Table 3:** Test results of some thermal properties of SPA composites and MPA composites.

GO Content (wt%)	T_m_ (°C)		T_c_ (°C)		_Δ_H_m_ (J/g)		T_d, 5%_ (°C)		HDT (°C)	
	SPA	MPA	SPA	MPA	SPA	MPA	SPA	MPA	SPA	MPA
0	296.51	296.51	270.98	270.98	44.94	44.94	424.09	424.09	77.8	77.8
0.5	294.69	295.96	264.06	273.48	35.10	47.80	412.41	420.44	84.7	83.9
1.0	295.42	294.67	270.99	269.28	36.45	39.89	406.67	412.42	99.3	97.4
1.5	290.99	293.56	265.79	270.39	27.23	43.78	405.12	413.67	120.7	107.5
2.0	293.80	292.65	269.28	271.49	44.03	40.85	400.20	408.73	144.2	117.3

**Table 4:** Main thermomechanical parameters of SPA and MPA composites with different GO content.

GO Content (wt%)	T_g_ (°C)		Tanδ at T_g_	
	SPA	MPA	SPA	MPA
0	106.64	106.64	0.24	0.24
0.50	118.21	104.68	0.48	0.38
1.00	118.03	102.57	0.28	0.24
1.50	114.34	102.92	0.67	0.44
2.00	118.97	104.38	0.29	0.37

**Table 5:** Mechanical properties of SPA composites and MPA composites.

GO Content (wt%)	Tensile Strength (Mpa)		Tensile Modulus (Gpa)		Elongation at Break (%)		Notched Impact Strength (KJ/m^2^)	
	SPA	MPA	SPA	MPA	SPA	MPA	SPA	MPA
0	70.86	70.86	14.70	14.70	170.47	170.47	6.42	6.42
0.5	77.39	73.79	14.92	14.83	31.02	21.87	9.39	8.49
1.0	81.86	75.16	16.45	15.42	16.47	36.07	12.18	10.55
1.5	96.63	78.47	18.07	16.51	11.67	45.27	13.56	10.60
2.0	108.89	80.57	16.26	15.26	14.23	21.75	16.45	11.88

## Data Availability

Not applicable.

## Sample Availability

Samples of the compounds are not available from the authors.
